# Comprehensive Characterization of Mutant *Pichia stipitis* Co-Fermenting Cellobiose and Xylose through Genomic and Transcriptomic Analyses

**DOI:** 10.4014/jmb.2209.09004

**Published:** 2022-10-14

**Authors:** Dae-Hwan Kim, Hyo-Jin Choi, Yu Rim Lee, Soo-Jung Kim, Sangmin Lee, Won-Heong Lee

**Affiliations:** 1 Department of Bioenergy Science and Technology, Chonnam National University, Gwangju 61186, Republic of Korea; 2 Department of Integrative Food, Bioscience and Biotechnology, Chonnam National University, Gwangju 61186, Republic of Korea; 3 Interdisciplinary Program of Agriculture and Life Science, Chonnam National University, Gwangju 61186, Republic of Korea; 4 Gwangju Bio/Energy R&D Center, Korea Institute of Energy Research, Gwangju 61003, Republic of Korea

**Keywords:** Mutant *Pichia stipitis*, cellobiose, xylose, whole-genome sequencing, RNA sequencing

## Abstract

The development of a yeast strain capable of fermenting mixed sugars efficiently is crucial for producing biofuels and value-added materials from cellulosic biomass. Previously, a mutant *Pichia stipitis* YN14 strain capable of co-fermenting xylose and cellobiose was developed through evolutionary engineering of the wild-type *P. stipitis* CBS6054 strain, which was incapable of cofermenting xylose and cellobiose. In this study, through genomic and transcriptomic analyses, we sought to investigate the reasons for the improved sugar metabolic performance of the mutant YN14 strain in comparison with the parental CBS6054 strain. Unfortunately, comparative wholegenome sequencing (WGS) showed no mutation in any of the genes involved in the cellobiose metabolism between the two strains. However, comparative RNA sequencing (RNA-seq) revealed that the YN14 strain had 101.2 times and 5.9 times higher expression levels of *HXT2.3* and *BGL2* genes involved in cellobiose metabolism, and 6.9 times and 75.9 times lower expression levels of *COX17* and *SOD2.2* genes involved in respiration, respectively, compared with the CBS6054 strain. This may explain how the YN14 strain enhanced cellobiose metabolic performance and shifted the direction of cellobiose metabolic flux from respiration to fermentation in the presence of cellobiose compared with the CBS6054 strain.

## Introduction

Various sugars derived from cellulosic biomass have been regarded as sustainable and environmentally friendly resources for microbial production of biofuels and biochemicals [1, 2]. However, these sugars are obtained in the form of mixed sugars that mainly contain glucose from the enzymatic saccharification of cellulosic biomass. This indicates that most microorganisms, including yeasts, sequentially utilize each sugar (glucose first and non-glucose sugars later) to produce biofuels and biochemicals because glucose and its metabolites inhibit the metabolism of non-glucose sugars (known as glucose repression) [3-5]. As a result of glucose repression, a decrease in product yield and productivity has been frequently observed during the fermentation of mixed sugars by yeasts. Therefore, a yeast strain that can efficiently ferment mixed sugars should be developed to effectively produce biofuels and biochemicals from cellulosic biomass [3, 4].

For efficient fermentation of mixed sugars, many studies have attempted to alleviate glucose repression in yeasts, such as *Saccharomyces cerevisiae*, a traditional host for bioethanol production [3, 4, 6]. However, complex and sophisticated mechanisms are involved in glucose repression, making it difficult to develop a yeast strain that can rapidly metabolize glucose and other sugars without glucose repression [3-6]. As an alternative to alleviating glucose repression, several studies have tried to develop a yeast strain that can ferment cellobiose, an intermediate product derived from the enzymatic saccharification of cellulose, to avoid glucose repression during the fermentation of mixed sugars derived from cellulosic biomass [3, 4, 7]. For example, engineered *S. cerevisiae* capable of fermenting xylose was further modified to ferment cellobiose by introducing cellobiose metabolic genes encoding cellobiose transporters and intracellular cellobiose-degrading enzymes. The resultant *S. cerevisiae* strain simultaneously fermented cellobiose and xylose and demonstrated markedly higher ethanol production yield and productivity than the parental strain that could sequentially ferment glucose and xylose [4, 8, 9]. However, the use of cellobiose-fermenting *S. cerevisiae* has resulted in unexpected problems during cellobiose fermentation, such as the accumulation of several oligomeric by-products by trans-glycosylation of intracellular cellobiose-degrading enzymes [9-11].

*Pichia stipitis*, a natural xylose-fermenting yeast, has also been used to produce biofuels from cellulosic biomass because it can metabolize various sugars derived from cellulosic biomass without the introduction of any heterologous genes [12-14]. However, wild-type *P. stipitis* exhibits a significantly slower cellobiose metabolic rate than xylose metabolic rate, indicating that it cannot efficiently ferment mixed sugars from cellulosic biomass without any improvement in cellobiose metabolism [15, 16]. Previously, a mutant *P. stipitis* YN14 strain was constructed by evolutionary engineering through serial subcultures of a wild-type *P. stipitis* CBS6054 strain in the presence of cellobiose. *P. stipitis* YN14 exhibited six-fold faster cellobiose consumption than the parental *P. stipitis* CBS6054 strain; therefore, the mutant strain could produce approximately two-fold higher amounts of ethanol than the parental strain through the simultaneous utilization of cellobiose and xylose during the fermentation of mixed sugars. In addition, the mutant YN14 strain was verified to show stable co-fermentation performance of cellobiose and xylose, even after serial subcultures in the presence of glucose [15]. These results suggest that the superior cellobiose metabolic performance of the mutant YN14 strain to that of the parental CBS6054 strain may be attributed to genetic mutations or changes in the expression levels of genes involved in cellobiose metabolism in the mutant strain. In the present study, we therefore aimed to identify the reasons for the improvement in cellobiose metabolism and the co-fermentation of cellobiose and xylose in the mutant YN14 strain in comparison with the parental CBS6054 strain by performing genomic and transcriptomic analyses.

## Materials and Methods

### Strains and Culture Conditions

Genomic and transcriptomic analyses were performed on the wild-type *P. stipitis* CBS6054 strain (haploid yeast) [13, 14] and the mutant *P. stipitis* YN14 strain [15]. Yeast extract-peptone (YP) medium (10 g/l of yeast extract and 20 g/l of Bacto-peptone, pH 6.7) containing 20 g/l of glucose (YPD20) was used for seed cultivation and pre-cultivation of *P. stipitis* strains. A single colony of each *P. stipitis* strain was picked from YPD agar plates and inoculated into 5 ml of YPD20. Seed cultivation was performed in 10-ml test tubes at 30°C and 250 rpm. After 24 h of seed cultivation, yeast cells were harvested and inoculated into 25 ml of YPD20. Pre-cultivation was performed in 125-ml flasks at 30°C and 250 rpm. YPD20 and YP media containing 30 g/l of cellobiose and 30 g/l of xylose (YPCX30) were used for the main cultivation of *P. stipitis* strains to obtain samples for genomic analysis and transcriptomic analysis, respectively. After 18 h of pre-cultivation, yeast cells were harvested, washed with sterilized water, and inoculated into 50 ml of YPD20 or YPCX30 at an initial cell concentration of 0.032 g/l. The main cultivation was performed in 250-ml flasks at 30°C and 90 rpm (micro-aerobic condition).

### Genomic Analysis

To compare the genetic mutations in the genome between the mutant *P. stipitis* YN14 strain and the parental *P. stipitis* CBS6054 strain, genomic analyses of the two strains were performed through whole-genome sequencing (WGS). Genomic analysis of the YN16 strain, another mutant *P. stipitis* strain exhibiting the same phenotype as the YN14 strain, was also performed to confirm the genetic mutation essential for enhanced cellobiose fermentation performance and co-utilization of cellobiose and xylose. Yeast cells in the exponential growth phase in YPD20 were sampled, and their genomic DNA was extracted using the Exgene Cell SV Mini Kit (GeneAll, Korea). WGS was performed using a commercial service (LAS Inc., Korea) by 300-bp, paired-end sequencing on an Illumina Miseq sequencer (Illumina Inc., USA). The sequence information of *P. stipitis* CBS6054 registered in the National Center for Biotechnology Information (NCBI) was used as the reference.

### Transcriptomic Analysis

To compare the changes in the expression levels of genes associated with cellobiose and xylose metabolism between the mutant *P. stipitis* YN14 strain and the parental *P. stipitis* CBS6054 strain, transcriptomic analyses of the two strains were performed by RNA sequencing (RNA-seq). The yeast cells cultured in YPCX30 were sampled when the xylose concentration was around 15 g/l (21 h of fermentation). Total RNA was extracted using the RNeasy Mini Kit (Qiagen Inc., Germany) after adjusting the concentration of the yeast cells to 1.3 g/l. RNA-seq was performed using a commercial service (LAS Inc.) by 150-bp, paired-end sequencing on an Illumina Miseq sequencer (Illumina Inc.). The mRNA sequence information of *P. stipitis* CBS6054 registered in the NCBI was used as the reference.

### Analytical Methods

Cell concentration was determined by measuring the optical density at 600 nm (UV-visible Biomate5 spectrophotometer, Thermo Fisher Scientific, USA) using a predetermined calibration curve. Cellobiose, xylose, and ethanol concentrations were determined using the Agilent Technologies 1200 Series HPLC system (Agilent, USA) equipped with a refractive index (RI) detector using a Rezex ROA-Organic Acid H^+^ (8%) column (Phenomenex Inc., USA). The column was eluted with 0.005 N of H_2_SO_4_ at a flow rate of 0.6 ml/min at 50°C.

## Results and Discussion

### Genomic Analysis of the Mutant and Parental Strains

In a previous study, the mutant YN14 strain exhibited six-fold higher consumption of cellobiose and co-fermentation of cellobiose and xylose compared with the parental CBS6054 strain [15]. Therefore, genomic analysis of YN14, YN16 (another mutant exhibiting the same phenotype as YN14), and CBS6054 strains was performed by WGS to identify the genetic mutations essential for enhanced cellobiose metabolism and co-fermentation of cellobiose and xylose in the mutant YN14 strain. The genomes of the three *P. stipitis* strains were extracted from the cells cultured in the presence of glucose (YPD20). The WGS results of the mutant strains compared with the parental strain are summarized in Table 1 (annotated genes with nonsynonymous mutations) and Table S1 (total mutated genes).

Compared with the CBS6054 strain, eight variants (five synonymous variants in four genes and three nonsynonymous variants in two genes) occurred in the protein-coding region in the genome of the mutant YN14 strain (Table S1). Of the three nonsynonymous variants, a single missense variant was found in *PICST_53564* (encoding a protein of unknown function) and double missense variants were found in *MUC1.10* (encoding a protein similar to Muc1p, a mucin-like protein) (Table 1, Table S1). Slightly different from the YN14 strain, the genome of the YN16 strain contained 11 variants (five synonymous variants in four genes and six nonsynonymous variants in four genes) in the protein-coding region in comparison with that of the CBS6054 strain (Table S1). The genes with six nonsynonymous variants were as follows: *PICST_65039* encoding a protein of unknown function (two missense variants), *PICST_53564* (one missense variant), *MUC1.10* (one missense variant), and *PPR2* encoding serine/threonine kinase (one frame-shift variant and one missense variant) (Table 1, Table S1).

When summarizing the WGS results for the YN14 and YN16 strains, the same mutation (substitution of the 260^th^ cytosine with thymine in the nucleotide sequence) was noted in *MUC1.10* in both mutant strains, resulting in a change in the amino acid sequence of Muc1.10p (substitution of the 87^th^ threonine with methionine). However, in contrast to our expectations, no mutation was observed in any gene involved in the metabolism of cellobiose or other carbons. As Muc1.10p is a protein similar to Muc1p, a cell surface glycoprotein involved in cell flocculation and pseudohyphal differentiation in yeasts [13, 17], mutation of *MUC1.10* is not thought to be associated with the enhanced cellobiose metabolism in the YN14 strain compared with the CBS6054 strain.

### Transcriptomic Analysis of the Mutant and Parental Strains

Based on the comparative WGS results between the mutant YN14 strain and the parental CBS6054 strain, the enhanced cellobiose metabolic performance of the YN14 strain was thought to be due to changes in the expression levels of cellobiose metabolic genes and not due to the mutation of cellobiose metabolic genes in the YN14 strain. Moreover, changes in the expression levels of other metabolic genes may have enabled the YN14 strain to simultaneously utilize cellobiose and xylose, in addition to improving its cellobiose metabolic rate. Therefore, transcriptomic analysis of the YN14 and CBS6054 strains was performed by RNA-seq to determine the proper reasons for the enhanced cellobiose metabolism and co-utilization of xylose and cellobiose in the YN14 strain.

**Fig. 1:**
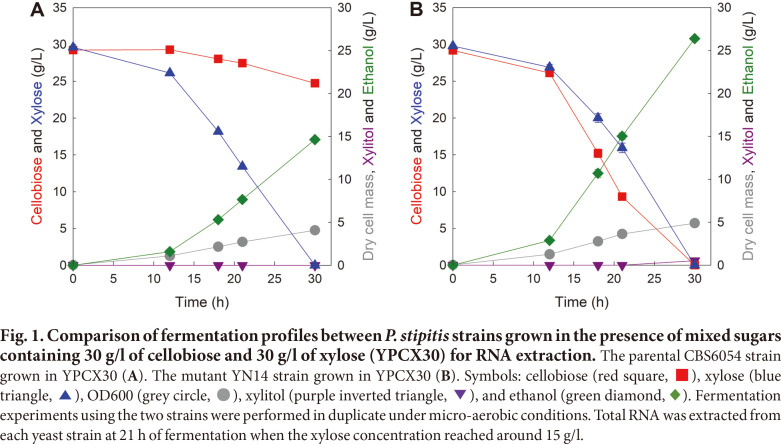
Fig. 1

To extract total RNA for transcriptomic analysis, the two *P. stipitis* strains were cultured in the presence of mixed sugars composed of 30 g/l of cellobiose and 30 g/l of xylose (YPCX30), as shown in Fig. 1. The fermentation profiles of the two strains were almost consistent with those reported in the previous study [15]. In the case of the parental CBS6054 strain, the entire xylose was consumed within 30 h of fermentation, while only 4.5 g/l of cellobiose was consumed during the same time. Therefore, only 14.6 g/l of ethanol was produced with a yield of 0.43 (g ethanol produced/g sugars consumed). Although the xylose consumption rate of the mutant YN14 strain was slightly slower than that of the parental CBS6054 strain, the YN14 strain co-consumed xylose and cellobiose for up to 30 h of fermentation. Consequently, 26.4 g/l of ethanol (1.8-fold higher than that produced by the CBS6054 strain) was produced with a yield of 0.45 (g ethanol/g sugars). In addition, the YN14 strain showed a 30%lower cell growth yield (0.08 g cells generated/g sugars consumed) than the CBS6054 strain (0.12 g cells/g sugars). These fermentation results indicate that the CBS6054 strain utilized xylose for ethanol production and cellobiose for cell growth, whereas the YN14 strain utilized both sugars for ethanol production. Thus, this finding suggests that the YN14 strain shifted the direction of cellobiose metabolism from respiration (for cell growth) to fermentation (for ethanol production) in contrast to the direction of cellobiose metabolism (only respiration) noted in the CBS6054 strain. The total RNA of each strain was extracted at 21 h of fermentation when the xylose concentration reached around 15 g/l, which was the time point at which the difference in cellobiose metabolism between the two strains was the highest during fermentation.

According to the RNA-seq results, of the total 5,818 genes encoding proteins in *P. stipitis*, the expression levels of 181 genes (approximately 3.1%) significantly differed between the YN14 strain and the CBS6054 strain [log_2_FC (fold change) value ≥ 1.0, *p*-value ≤ 0.05]. Of these, the expression levels of 96 genes were upregulated, while those of 85 genes were downregulated. The results of transcriptomic analysis between the two strains are summarized in Table S2. In particular, the genes involved in central carbon metabolism (such as cellobiose transport, xylose transport, glycolysis, pentose phosphate and fermentative pathways, and respiration) and other cellular functions (such as stress response and ion metabolism) are selectively summarized in Table 2.

### Comparison of the Expression Levels of Cellobiose Metabolic Genes and Glycolytic Genes between the Mutant and Parental Strains

**Fig. 2:**
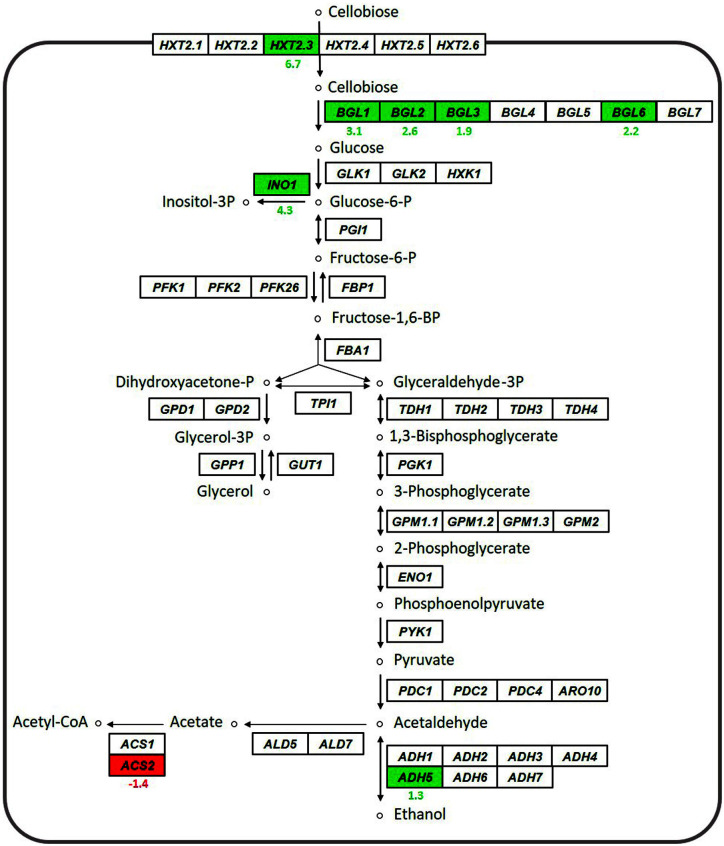
Comparison of expression patterns of genes involved in cellobiose transport, intracellular cellobiose degradation, glycolysis, and fermentative pathway between the mutant *P. stipitis* YN14 strain and the parental *P. stipitis* CBS6054 strain grown in YPCX30. Transcriptomes that significantly increased and decreased in the YN14 strain in contrast to the CBS6054 strain [log_2_FC (fold change) value ≥ 1.0, *p*-value ≤ 0.05] are shown in green and red boxes, respectively. The differences in gene expression levels between the two strains are expressed as the log_2_FC value.

Fig. 2 shows the comparison of the expression patterns of genes involved in cellobiose transport, intracellular degradation of cellobiose, glycolysis, and fermentative pathway between the YN14 strain and the CBS6054 strain (log_2_FC ≥ 1.0, P ≤ 0.05). Overall, except for genes involved in cellobiose transport and intracellular degradation of cellobiose, the expression levels of most genes involved in glycolysis and fermentative pathways did not differ significantly between the two strains. As glycolytic genes *in* *P. stipitis* are known to be expressed at sufficient levels regardless of culture conditions (*e.g.*, type of sugar or degree of aeration) [14, 18], it is plausible that no significant difference existed in the expression levels of genes related to glycolysis between the two strains.

In *P. stipitis*, six *HXT* genes (*HXT2.1–2.6*) and seven *BGL* genes (*BGL1–7*) encode cellobiose transporters and intracellular β-glucosidases, respectively [13, 14, 18]. Among the *HXT* genes, the expression level of only *HXT2.3* was noticeably higher in the YN14 strain than in the CBS6054 strain (log_2_FC = 6.7), indicating that strongly induced expression of *HXT2.3* may be one of the main reasons for the enhanced cellobiose metabolism in the YN14 strain. Although other *HXT* genes (such as *HXT2.5* and *HXT2.6*) also showed significantly increased expression levels in the YN14 strain than in the CBS6054 strain (log_2_FC for *HXT2.5* and *HXT2.6* = 7.8 and 8.8, respectively; *p* = 0.07 and 0.23, respectively; data not shown in Tables), they were not considered to be the key contributors improving cellobiose metabolism in the YN14 strain because their expression levels were more than 10 times lower than that of *HXT2.3* in the YN14 strain (expression level of *HXT2.5* and *HXT2.6* = 51.1 and 29.0, respectively, in YN14; data not shown in Tables). Among the *BGL* genes, four genes (*BGL1–3* and *BGL6*) showed significantly higher expression levels in the YN14 strain than in the CBS6054 strain (log_2_FC = 1.9 to 3.1). As the expression level of *BGL2* was more than three-fold higher than that of the other three *BGL* genes (*BGL1, 2* and *6*) in both YN14 and CBS6054 strains (Table 2), a significant increase in the expression level of *BGL2* may be another reason for the enhanced cellobiose metabolism in the YN14 strain. Interestingly, the expression levels of *HXT2.4* and *BGL5*, which are known to play major roles in cellobiose metabolism in *P. stipitis* [14, 19], did not differ significantly between the YN14 and CBS6054 strains (log_2_FC for *HXT2.4* and *BGL5* = 1.1 and 1.0, respectively; *p* = 0.17 and 0.20, respectively; data not shown in Tables). As the expression levels of *HXT2.4* and *BGL5* have been reported to be considerably higher than those of other *HXT* and *BGL* genes in *P. stipitis* in the presence of cellobiose [14, 19], their expression levels in the YN14 strain may not have increased significantly compared with those in the CBS6054 strain.

In addition to *HXT2.3* and *BGL2*, considerable differences in the expression levels of *ADH5* and *ACS2* were found between the YN14 and CBS6054 strains (log_2_FC for *ADH5* and *ACS2* = 1.3 and −1.4, respectively). As Adh5p is an alcohol dehydrogenase generating ethanol for fermentation and Acs2p is an acyl-CoA synthase generating acetyl-CoA for fatty acid biosynthesis [13, 14], the YN14 strain may have promoted and limited the expression of the *ADH5* and *ACS2*, respectively, to redirect cellobiose metabolism toward fermentation rather than cell growth-related metabolism. Interestingly, the expression level of *INO1* (encoding inositol-3-phosphate synthase) was remarkably higher in the YN14 strain than in the CBS6054 strain (log_2_FC = 4.3), although this gene is not considered to be directly related to fermentation or respiration [20]. Previously, supplementation of inositol during ethanol fermentation was found to improve the tolerance of yeast cells to high concentrations of ethanol [21]. The YN14 strain produced around 1.8-fold higher amounts of ethanol than the CBS6054 strain during the fermentation of mixed sugars (Fig. 1); therefore, the YN14 strain may have strongly induced the expression of genes involved in inositol synthesis, such as *INO1*, to alleviate the stress caused by the increased production of ethanol, in contrast to the CBS6054 strain.

### Comparison of the Expression Levels of Xylose Metabolic Genes between the Mutant and Parental Strains

**Fig. 3:**
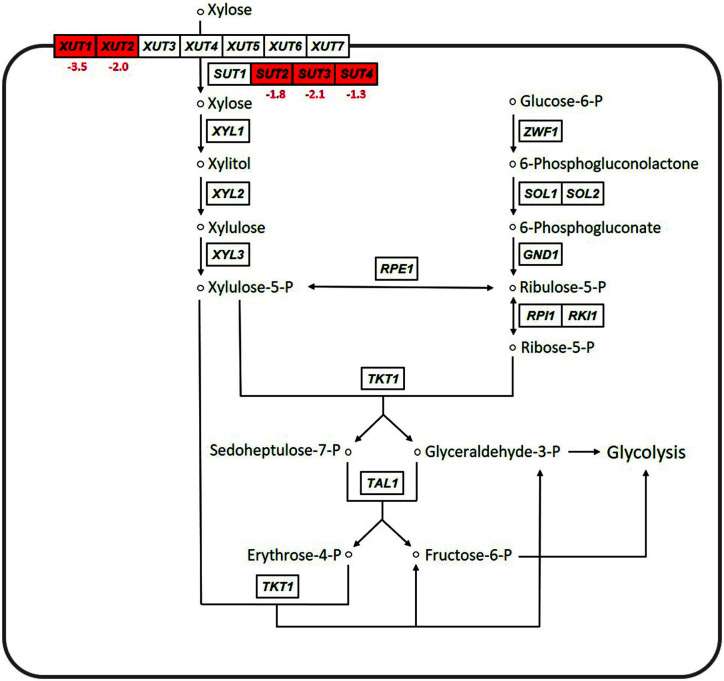
Comparison of expression patterns of genes involved in xylose transport and pentose phosphate pathway between the mutant YN14 strain and the parental CBS6054 strain grown in YPCX30. Transcriptomes that significantly increased and decreased in YN14 in contrast to CBS6054 (log_2_FC ≥ 1.0, *p* ≤ 0.05) are shown in green and red boxes, respectively, with log_2_FC values.

Fig. 3 compares the expression patterns of genes involved in xylose transport and pentose phosphate pathway between the YN14 and CBS6054 strains (log_2_FC ≥ 1.0, *p* ≤ 0.05). Similar to the expression patterns of glycolytic genes shown in Fig. 2, except for the genes involved in xylose transport, significant differences in the expression levels of genes involved in pentose phosphate pathway, including those involved in the conversion of xylose to xylulose-5-phosphate, were not observed between the two strains.

In *P. stipitis*, seven *XUT* genes (*XUT1–7*) and four *SUT* genes (*SUT1–4*) are known to encode xylose transporters [13, 14, 18]. Interestingly, the expression levels of five of these genes (*XUT1–2* and *SUT2–4*) were significantly lower in the YN14 strain than in the CB6054 strain (log_2_FC = −3.5 to −1.3), although the YN14 strain exhibited a similar xylose consumption rate to the CBS6054 strain (Fig. 1). Several previous studies evaluating the sugar transport efficiencies of xylose transporters in *P. stipitis* have reported that Sut1p exhibits considerably higher xylose transport activity than other transporters [22-25], suggesting that *SUT1* plays an important role in xylose transport in *P. stipitis* during xylose fermentation. As the expression level of *SUT1* was significantly higher than that of other xylose transporter genes in both strains based on RNA-seq results (expression level of *SUT1* in CBS6054 and YN14 = 1482.9 and 1871.2, respectively; log_2_FC = 0.34; *p* = 0.67; data not shown in Tables), the reduction of xylose metabolism in the YN14 strain may not have occurred because of the stable expression of *SUT1* despite the reduced expression of five transporter genes (*XUT1–2* and *SUT2–4*). Therefore, the YN14 strain may have exhibited similar xylose fermentation performance to the CBS6054 strain. In addition, the spatial constraint of the cell membrane due to the increased expression of cellobiose transporters may have resulted in the reduced expression of xylose transporter genes.

In the case of xylose metabolic genes, the expression levels of *XYL1* (encoding xylose reductase) and *XYL2* (encoding xylitol dehydrogenase) were slightly lower in the YN14 strain than in the CBS6054 strain (log_2_FC for *XYL1* and *XYL2* = −1.17 and −1.28, respectively; *p* = 0.06 and 1.12, respectively; data not shown in Tables). However, the expression level of *XYL3* (encoding xylulokinase) was almost the same in both strains (log_2_FC = 0.02, *p* = 0.98, data not shown in Tables). Because *P. stipitis* is known to significantly elevate the expression levels of genes involved in pentose phosphate pathway and xylose assimilation in the presence of xylose [13, 14, 18], a slight decrease in the expression levels of *XYL1* and *XYL2* may not have disturbed the xylose metabolic performance of the YN14 strain, thereby allowing the YN14 strain to co-ferment xylose and cellobiose.

### Comparison of the Expression Levels of Respiratory Genes between the Mutant and Parental Strains

**Fig. 4:**
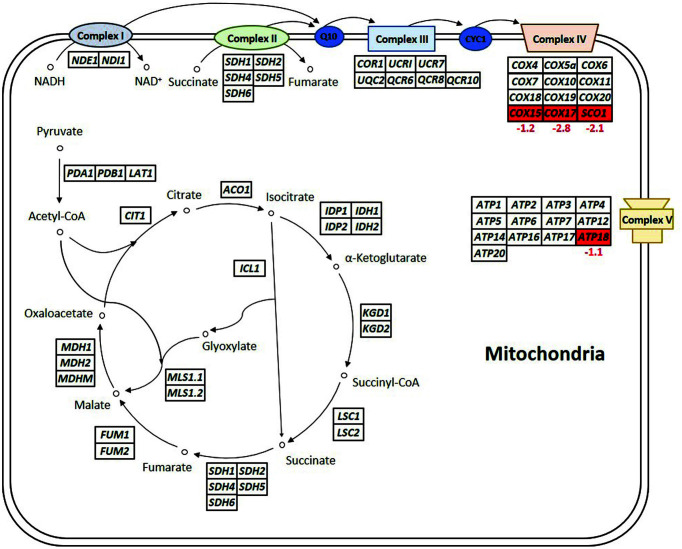
Comparison of expression patterns of genes involved in mitochondrial functions (such as tricarboxylic acid cycle, glyoxylate cycle, electron transport chain, and oxidative phosphorylation) between the mutant YN14 strain and the parental CBS6054 strain grown in YPCX30. Transcriptomes that significantly increased and decreased in YN14 in contrast to CBS6054 (log_2_FC ≥ 1.0, *p* ≤ 0.05) are shown in green and red boxes, respectively, with log_2_FC values.

Fig. 4 shows the comparison of the expression patterns of genes involved in the respiration process in the mitochondria, such as the tricarboxylic acid cycle, electron transport chain, and oxidative phosphorylation, between the YN14 and CBS6054 strains (log_2_FC ≥ 1.0, *p* ≤ 0.05). Initially, the expression levels of many genes involved in respiration were considered to differ significantly between the YN14 and CBS6054 strains because the YN14 strain preferred fermentation (ethanol production) rather than respiration (cell growth) when metabolizing cellobiose. However, except for four genes involved in the electron transport chain and oxidative phosphorylation (*COX15*, *COX17*, *SCO1* and *ATP18*), the expression levels of other respiratory genes did not differ significantly between the YN14 and CBS6054 strains.

Most respiratory genes are known to be expressed at lower levels in *P. stipitis* under micro-aerobic conditions than under aerobic conditions [14]. When fermentation was performed under micro-aerobic conditions (Fig. 1), the CBS6054 strain exhibited an extremely slow cellobiose consumption rate, suggesting that the CBS6054 strain could utilize cellobiose only for respiration despite the low expression levels of respiratory genes under micro-aerobic conditions. In contrast, the YN14 strain exhibited a remarkably fast cellobiose consumption rate, suggesting that the YN14 strain could easily switch the direction of cellobiose metabolism from respiration to fermentation by reducing the expression of a few genes essential for respiration because most respiratory genes were already expressed at low levels under micro-aerobic conditions.

Consequently, three genes (*COX15*, *COX17* and *SCO1*) encoding the components of cytochrome c oxidase (the last and rate-limiting enzyme in electron transport chain) and one gene (*ATP18*) encoding a subunit of ATP synthase (the energy generator in oxidative phosphorylation) showed considerably lower expression levels in the YN14 strain than in the CBS6054 strain (log_2_FC = −2.8 to −1.1). Cox17p and Sco1p have been reported to be necessary for the insertion of copper ions into catalytic subunits of cytochrome c oxidase, and Cox15p is required for the biosynthesis of heme A, a cofactor of cytochrome c oxidase in yeasts [26-29]. Moreover, Atp18p is required for the stable expression of ATP synthase in yeasts [30]. As the deletion of *COX17* or *SCO1* was found to cause a respiratory-deficient phenotype in yeast cells [26, 27] and the expression levels of *COX17* and *SCO1* were observed to be more than two-fold higher than that of *COX15* in the CBS6054 strain (Table 2), the reduced expression of *COX17* and *SCO1* may be the key factor associated with the redirection of cellobiose metabolic flux from respiration to fermentation in the YN14 strain. In addition, the inhibition of cytochrome c oxidase could diminish ATP generation in the mitochondria of yeast cells [31], suggesting that the reduced expression of *COX17* and *SCO1* could affect the expression of *ATP18*, the essential subunit gene for ATP synthase, in the YN14 strain.

### Comparison of the Expression Levels of Stress-Responsive Genes between the Mutant and Parental Strains

**Fig. 5:**
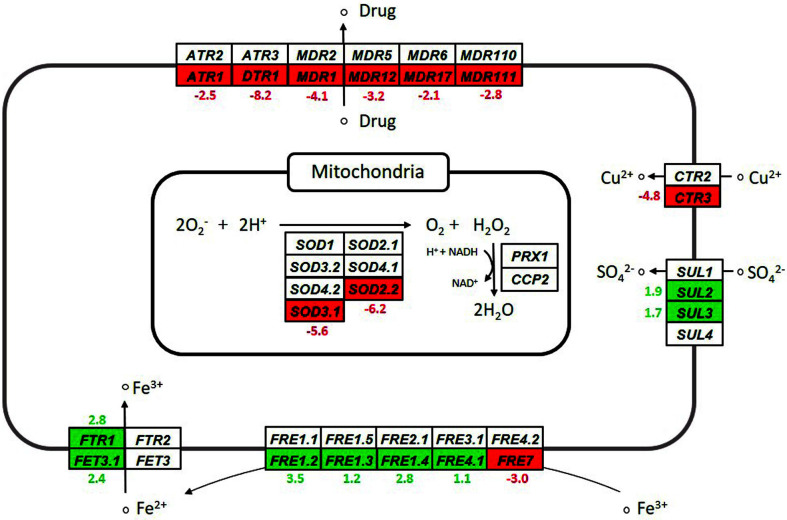
Comparison of expression patterns of genes involved in stress response (such as chemical and oxidative stress) and ion assimilation between the mutant YN14 strain and the parental CBS6054 strain grown in YPCX30. Transcriptomes that significantly increased and decreased in YN14 in contrast to CBS6054 (log_2_FC ≥ 1.0, *p* ≤ 0.05) are shown in green and red boxes, respectively, with log_2_FC values.

In addition to the changes in the expression levels of genes involved in the respiratory process, the expression levels of the genes involved in several cellular functions related to respiration may have been modulated in the YN14 strain in comparison with the CBS6054 strain. Fig. 5 compares the expression patterns of genes involved in other cellular functions related to respiration, such as ion assimilation and oxidative and chemical stress responses, between the YN14 and CBS6054 strains (log_2_FC ≥ 1.0, *p* ≤ 0.05).

Reactive oxygen species (ROS), which are known to damage yeast cell components and even trigger apoptosis, are generated during respiration in the mitochondria [32, 33]. To scavenge ROS, yeasts are known to express several types of enzyme, such as superoxide dismutase and peroxidase [32, 33]. Of the several genes involved in ROS detoxification in *P. stipitis*, the expression levels of *SOD2.2* (encoding Cu/Zn superoxide dismutase) and *SOD3.1* (encoding Mn superoxide dismutase) were significantly lower in the YN14 strain than in the CBS6054 strain (log_2_FC for *SOD2.2* and *SOD3.1* = −6.2 and −5.6, respectively). Because of the decrease in the expression levels of genes involved in electron transport and oxidative phosphorylation, such as *COX17*, *SCO1*, and *ATP18*, the generation of ROS may have been considerably lower in the YN14 strain than in the CBS6054 strain, thereby limiting unnecessary expression of *SOD* genes associated with ROS detoxification in the YN14 strain.

According to previous studies, ion metabolism is associated with respiration and ROS degradation in yeasts [26, 27, 29, 34-36]. Several components of cytochrome c oxidase, including Cox17p and Sco1p, not only require copper ions as a cofactor but also participate in copper metabolism [26, 27, 29, 36]. The mutant YN14 strain showed significantly reduced expression levels of *COX17* and *SCO1*; this may have resulted in a significantly lower expression level of the copper transporter gene (*CTR3*) in the YN14 strain than in the CBS6054 strain (log2FC = -4.8). Supplementation of iron has been reported to improve resistance to ROS in *S. cerevisiae* lacking superoxide dismutase [35], suggesting that the YN14 strain exhibiting significantly reduced *SOD* expression may have promoted iron metabolism, particularly iron influx into cells. Consequently, the expression levels of genes (*FTR1* and *FET3.1*) encoding iron permease and multicopper oxidase, which form the permease-oxidase complex for iron transport, were significantly higher in the YN14 strain than in the CBS6054 strain (log_2_FC for *FTR1* and *FET3.1* = 2.8 and 2.4, respectively). The expression levels of four genes (*FRE1.2–1.4* and *FRE4.1*) encoding ferric reductases, the enzymes converting Fe^3+^ to Fe^2+^, were also significantly higher in the YN14 strain than in the CBS6054 strain (log_2_FC = 1.1 to 3.5). As Fe^2+^ is the substrate for the permease-oxidase complex [34, 36, 37], an increase in the expression levels of *FRE* genes may have been required for a sufficient supply of Fe^2+^ to the iron transporter complex. In addition, SO_4_^2-^ is an essential ion in yeasts for switching the sugar metabolic flux from oxidative metabolism to fermentative metabolism [38]. This may be the reason why the expression levels of the genes encoding sulfate transporter (*SUL2–3*) were considerably higher in the YN14 strain than in the CBS6054 strain (log_2_FC for *SUL2* and *SUL3* = 1.9 and 1.7, respectively).

As in the case of *INO1* expression, the YN14 strain may be subjected to stress as a result of excessive ethanol formation. This suggests that the YN14 strain could induce the expression of genes related to chemical stress response [39]. However, in contrast to our expectations, the expression levels of several genes encoding multidrug resistance proteins for chemical stress response (such as *ATR1*, *DTR1*, and *MDR1–111*) were significantly lower in the YN14 strain than in the CBS6054 strain. In particular, the expression level of *DTR1* (encoding dityrosine transporter) was 18-fold higher than that of other stress response genes in the CBS6054 strain (expression levels of *DTR1* and other genes = 1410.64, and 4.99 to 76.48, respectively); however, the expression of *DTR1* was the most severely limited in the YN14 strain compared with that of other stress response genes, as shown in Table 2 (log_2_FC for *DTR1* = −8.2, log_2_FC for other genes = −4.1 to −2.1). The expression of *DTR1* is known to be induced when the yeast cells face harsh conditions, *e.g.*, in the presence of a non-fermentable carbon source [40]. As the mutant YN14 strain evolved to rapidly ferment cellobiose, a sugar that the parental CBS6054 strain could not ferment at all, it may be plausible that the expression level of *DTR1* was significantly lower in the YN14 strain than in the CBS6054 strain. On the other hand, other stress response proteins are known to be drug-efflux pumps excreting cytotoxic chemicals, as a type of ATP-binding cassette (ABC) transporter [39, 41]. As mentioned above, the YN14 strain switched the metabolic flux of cellobiose from respiration to fermentation. Thus, compared with the CBS6054 strain, the YN14 strain may not have generated sufficient amounts of ATP. Consequently, the expression of drug-efflux pumps may have been limited in the YN14 strain to avoid wasting energy.

In this study, we assessed the appropriate reasons for the enhanced cellobiose metabolic performance and co-fermentation of cellobiose and xylose in the mutant *P. stipitis* YN14 strain compared with the parental *P. stipitis* CBS6054 strain. Although comparative genomic analysis between the two strains did not yield any significant results, comparative transcriptomic analysis between the two strains indicated a significant increase in the expression levels of cellobiose metabolic genes (such as *HXT2.3* and *BGL2*) without a significant change in the expression levels of xylose metabolic genes (such as *XYL1*, *XYL2* and *XYL3*) may be responsible for the improved cellobiose metabolism and co-fermentation of cellobiose and xylose in the YN14 strain in comparison with the CBS6054 strain. Comparative transcriptomic analysis also indicated that significant changes in the expression levels of respiratory or respiratory-related genes (such as *COX17*, *SCO1*, *SOD2.2*, *SOD3.1*, *CTR3*, *FTR1*, and *FET3.1*) may be responsible for the redirection of cellobiose metabolic flux from oxidative metabolism to fermentative metabolism in the YN14 strain. Genetic information on the mutant *P. stipitis* strain co-fermenting cellobiose and xylose may be useful in developing a yeast strain suitable for producing biofuels and biochemicals from cellulosic biomass.

## Supplemental Materials

Supplementary data for this paper are available on-line only at http://jmb.or.kr.

## Figures and Tables

**Table 1:** Identification of genetic mutations in the protein-coding sequence (CDS) region in the genome of the two mutant *P. stipitis* strains, YN14 and YN16, in comparison with that of the parental *P. stipitis* CBS6054 strain.

Gene with nonsynonymous mutation	Changes in YN14		Changes in YN16	

	Nucleotide	Amino acid	Nucleotide	Amino acid
*MUC1.10* (encoding protein with similarity to mucin-like protein)	A137C	His46Pro		
	C260T	Thr87Met	C260T	Thr87Met
*PPR2* (encoding serine/threonine kinase)			ACAAATCTGC 348–357del (deletion)	Gln117fs (frame-shift)
			G364A	Val122Ile
Total mutations in CDS	8		11	
Total mutated genes in CDS	2		4	

**Table 2:** Upregulated or downregulated transcriptomes in the mutant *P. stipitis* YN14 strain in comparison with the parental *P. stipitis* CBS6054 strain [log_2_FC (fold change) value ≥ 1.0, *p* ≤ 0.05].

	Genes	Description of the genes	Type of metabolism	Log_2_FC	Expression levels	

					CBS6054	YN14
Up-regulated	*HXT2.3*	Probable hexose transporter	Cellobiose metabolism	6.7	7.28	736.78
	*BGL1*	β-glucosidase	Cellobiose metabolism	3.1	41.53	354.99
	*BGL2*	β-glucosidase	Cellobiose metabolism	2.6	282.21	1659.38
	*BGL6*	β-glucosidase	Cellobiose metabolism	2.2	3.47	16.33
	*BGL3*	β-glucosidase	Cellobiose metabolism	1.9	99.90	368.06
	*ADH5*	Alcohol hydrogenase	Ethanol fermentation	1.3	87.35	211.76
	*INO1*	Inositol-3-phosphate synthase	Chemical stress response	4.3	77.83	1575.51
	*FRE1.2*	Ferric reductase	Metal ion assimilation	3.5	3.59	39.50
	*FTR1*	Iron permease	Metal ion assimilation	2.8	242.83	1715.75
	*FRE1.4*	Ferric reductase	Metal ion assimilation	2.8	40.74	279.75
	*FET3.1*	Multicopper oxidase	Metal ion assimilation	2.4	267.80	1454.92
	*FRE1.3*	Ferric reductase	Metal ion assimilation	1.2	11.15	25.22
	*FRE4.1*	Ferric reductase	Metal ion assimilation	1.1	12.82	27.42
	*SUL2*	High-affinity sulfate permease	Anion assimilation	1.9	4.40	16.76
	*SUL3*	Putative sulfate transporter	Anion assimilation	1.7	11.45	37.26
Down-regulated	*XUT1*	Sugar transporter	Xylose metabolism	−3.5	576.00	50.39
	*SUT3*	Sugar transporter	Xylose metabolism	−2.1	62.57	15.02
	*XUT2*	Sugar transporter	Xylose metabolism	−2.0	19.82	4.84
	*SUT2*	Sugar transporter	Xylose metabolism	−1.8	70.09	20.53
	*SUT4*	Sugar transporter	Xylose metabolism	−1.3	750.51	301.85
	*ACS2*	Acyl-CoA synthetase 2	Fatty acid metabolism	−1.4	209.06	78.66
	*COX17*	Cytochrome c oxidase assembly protein	Respiration	−2.8	1031.50	149.37
	*SCO1*	Putative cytochrome c oxidase assembly protein	Respiration	−2.1	1195.23	279.00
	*COX15*	Cytochrome c oxidase assembly protein	Respiration	−1.2	454.80	191.33
	*ATP18*	Subunit of mitochondrial ATP synthase	Respiration	−1.1	2213.29	1059.02
	*SOD2.2*	Cu/Zn superoxide dismutase	Oxidative stress response	−6.2	5562.32	73.25
	*SOD3.1*	Mn superoxide dismutase	Oxidative stress response	−5.6	1023.78	21.69
	*CTR3*	Copper transporter	Metal ion assimilation	−4.8	6210.51	225.13
	*FRE7*	Ferric reductase	Metal ion assimilation	−3.0	878.93	110.48
	*DTR1*	Dityrosine transporter	Chemical stress response	−8.2	1410.64	4.66
	*MDR1*	Multidrug resistance transporter	Chemical stress response	−4.1	76.48	4.59
	*MDR12*	Multidrug resistance protein	Chemical stress response	−3.2	13.53	1.50
	*MDR111*	Putative transporter C530	Chemical stress response	−2.8	13.10	1.92
	*ATR1*	Multidrug resistance transporter	Chemical stress response	−2.5	4.99	0.88
	*MDR17*	Multidrug resistance protein 7	Chemical stress response	−2.1	24.28	5.57

Genes involved in central carbon metabolism (cellobiose transport, xylose transport, glycolysis, pentose phosphate pathway, fermentative pathway, and respiration) and other cellular functions (stress response and ion assimilation) are selectively listed.
